# Crystal structure of 2-benzamido-*N*-(2,2-di­eth­oxy­eth­yl)benzamide

**DOI:** 10.1107/S2056989015003370

**Published:** 2015-02-28

**Authors:** Abdelaaziz Ouahrouch, Moha Taourirte, Hassan B. Lazrek, Joachim W. Engels, Michael Bolte

**Affiliations:** a Laboratory of Bioorganic and Macromolecular Chemistry, Department of Chemistry, Faculty of Sciences and Technology Guéliz (FSTG), BP 549, Marrakech, Morocco; b Laboratory of Biomolecular and Medicinal Chemistry, Department of Chemistry, Faculty of Sciences Semlalia, Marrakech, Morocco; c Institut für Organische Chemie und Chemische Biologie, Goethe-Universität Frankfurt, Max-von-Laue-Strasse 7, 60438 Frankfurt am Main, Germany; d Institut für Anorganische und Analytische Chemie, Goethe-Universität Frankfurt, Max-von-Laue-Strasse 7, 60438 Frankfurt am Main, Germany

**Keywords:** crystal structure, N—H⋯O hydrogen bonds

## Abstract

In the title compound, C_20_H_24_N_2_O_4_, both peptide bonds adopt a *trans* configuration with respect to the —N—H and —C=O groups. The dihedral angle between the aromatic rings is 53.58 (4)°. The mol­ecular conformation is stabilized by an intra­molecular N—H⋯O hydrogen bond. The crystal packing is characterized by zigzag chains of N—H⋯O hydrogen-bonded mol­ecules running along the *b-*axis direction.

## Related literature   

For the synthesis of the title compound, see: Xingwen *et al.* (2007 ▸); Chandrika *et al.* (2008 ▸). Compounds with quinazoline scaffolds are of biological importance due to their pharmacological activities such as anti­microbial (Jantova *et al.*, 2004 ▸; Shi *et al.*, 2013 ▸), anti­tumorigenic (Kubo *et al.*, 2005 ▸), anti­fungal (Dandia *et al.*, 2005 ▸), anti­hyperglycemic (Ram *et al.*, 2003 ▸), anti-inflammatory (Gineinah *et al.*, 2002 ▸; Baba *et al.*, 1996 ▸), anti­tumor (Forsch *et al.*, 2002 ▸) and protein kinase inhibitor (Levitzky, 2003 ▸).
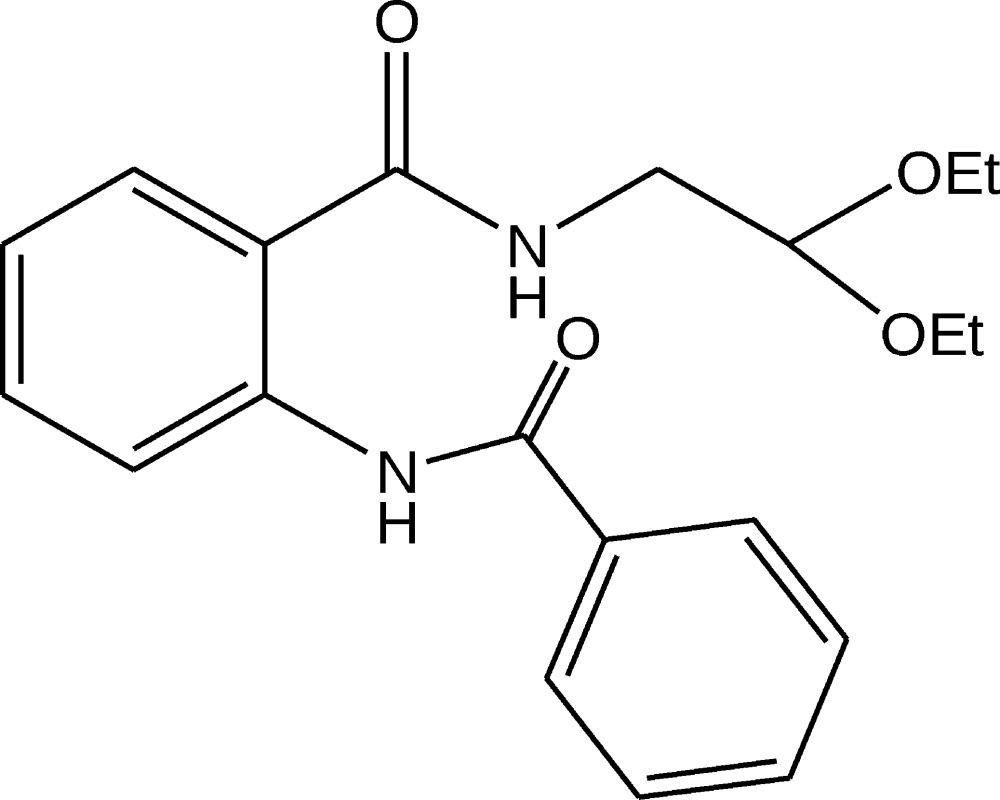


## Experimental   

### Crystal data  

C_20_H_24_N_2_O_4_
*M* *_r_* = 356.41
Monoclinic, 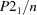

*a* = 8.4901 (4) Å
*b* = 14.2281 (7) Å
*c* = 15.3864 (7) Å
β = 98.659 (4)°
*V* = 1837.46 (15) Å^3^
*Z* = 4
Mo *K*α radiation
μ = 0.09 mm^−1^
*T* = 173 K
0.36 × 0.35 × 0.32 mm

### Data collection  

Stoe IPDS II two-circle diffractometer
Absorption correction: multi-scan (*X-AREA*; Stoe & Cie, 2001 ▸) *T*_min_ = 0.874, *T*_max_ = 0.892
56243 measured reflections
5148 independent reflections
4676 reflections with *I* > 2σ(*I*)
*R*_int_ = 0.065

### Refinement  

*R*[*F* ^2^ > 2σ(*F* ^2^)] = 0.041
*wR*(*F* ^2^) = 0.110
*S* = 1.08
5148 reflections
244 parameters
H atoms treated by a mixture of independent and constrained refinement
Δρ_max_ = 0.35 e Å^−3^
Δρ_min_ = −0.18 e Å^−3^

### 

Data collection: *X-AREA* (Stoe & Cie, 2001 ▸); cell refinement: *X-AREA*; data reduction: *X-AREA*; program(s) used to solve structure: *SHELXS97* (Sheldrick, 2008 ▸); program(s) used to refine structure: *SHELXL97* (Sheldrick, 2008 ▸); molecular graphics: *XP* (Sheldrick, 2008 ▸) and *PLATON* (Spek, 2009 ▸); software used to prepare material for publication: *SHELXL97* and *publCIF* (Westrip, 2010 ▸).

## Supplementary Material

Crystal structure: contains datablock(s) I, global. DOI: 10.1107/S2056989015003370/lh5750sup1.cif

Structure factors: contains datablock(s) I. DOI: 10.1107/S2056989015003370/lh5750Isup2.hkl

Supporting information file. DOI: 10.1107/S2056989015003370/lh5750Isup3.cml

**Fig. 1.**
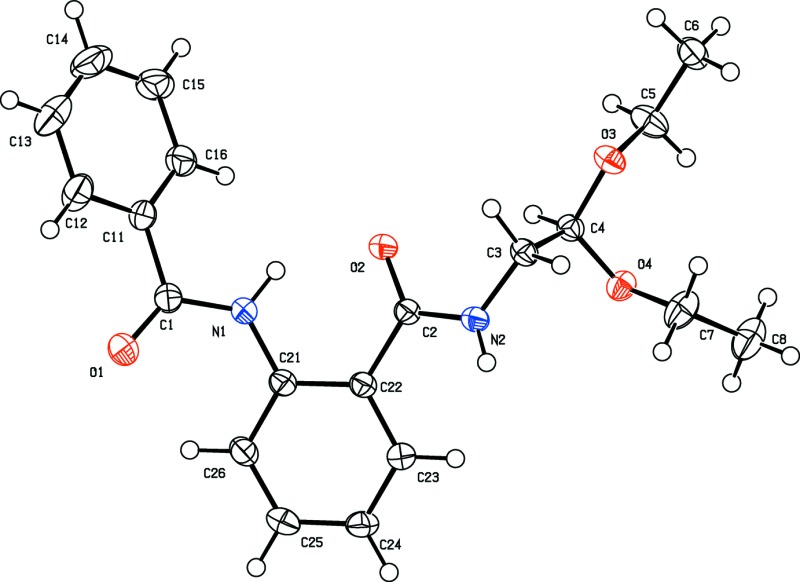
A perspective view of the title compound, showing the atom-numbering scheme. Displacement ellipsoids are drawn at the 50% probability level and H atoms are shown as small spheres of arbitrary radii.

. DOI: 10.1107/S2056989015003370/lh5750fig1.tif
A perspective view of the title compound, showing the atom-numbering scheme. Displacement ellipsoids are drawn at the 50% probability level and H atoms are shown as small spheres of arbitrary radii.

**Fig. 2.**
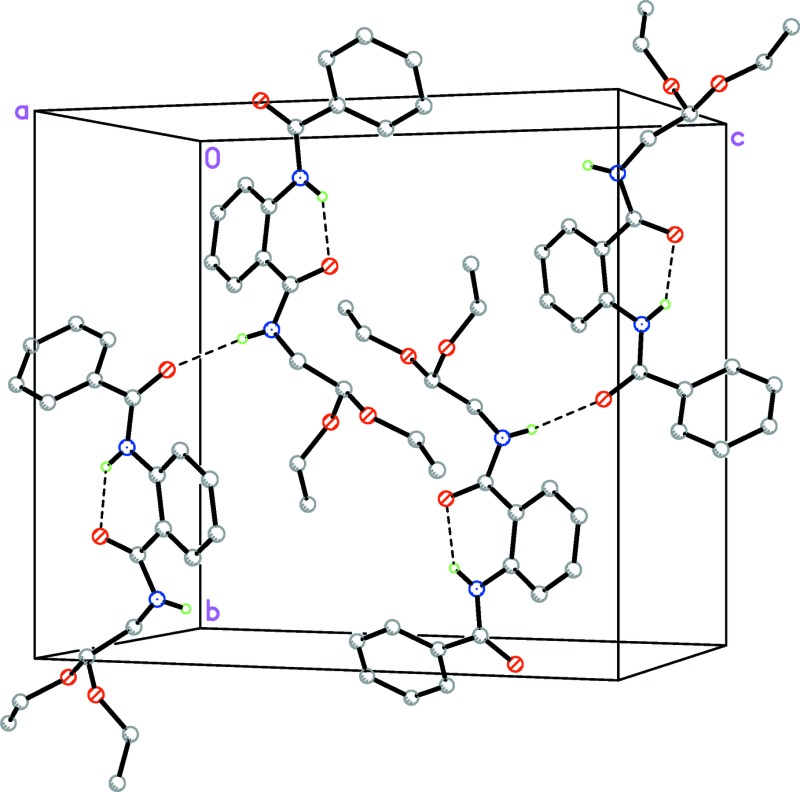
Crystal packing of the title compound. Hydrogen atoms bonded to C are omitted for clarity. Hydrogen bonds are shown as dashed lines.

. DOI: 10.1107/S2056989015003370/lh5750fig2.tif
Crystal packing of the title compound. Hydrogen atoms bonded to C are omitted for clarity. Hydrogen bonds are shown as dashed lines.

CCDC reference: 1050003

Additional supporting information:  crystallographic information; 3D view; checkCIF report

## Figures and Tables

**Table 1:** Hydrogen-bond geometry (, )

*D*H*A*	*D*H	H*A*	*D* *A*	*D*H*A*
N1H1O2	0.886(16)	1.914(15)	2.6434(11)	138.6(14)
N2H2O1^i^	0.848(15)	2.132(15)	2.9338(12)	157.6(13)

Symmetry code: (i) 
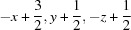
.

## supplementary crystallographic information

### S1.1. Synthesis and crystallization

The compound 2-(phenyl­carbonyl­amino)-N-(2,2-di­eth­oxy­ethyl)­benzamide was synthesized by reaction of anthranilic acid with benzoyl chloride in dry pyridine at 273-278 K for 4 hours to obtain 2-(3-chloro­phenyl)-benzo[d][1,3]oxazin-4-one in high yields (Xingwen *et al.*, 2007, Chandrika *et al.*, 2008). The obtained inter­mediate was treated with 1.5 equivalent of 2,2-di­eth­oxy­ethanamine under microwave irradiation for 2 min. The resulting residue was purified by column chromatography using ethyl acetate/hexane (80/20) as eluent. The crystal suitable for X-ray analysis was obtained by slow evaporation of a solution of the title compound in ethyl acetate/hexane 4:1 v/v (Melting point: 362-363 K).

### S1.2. Refinement

Hydrogen atoms were initially located in difference Fourier maps. Subsequently, H atoms bonded to C atoms were refined using a riding model, with tertiary C—H = 1.0 Å, methyl C—H = 0.98 Å, secondary C—H = 0.99 Å and aromatic C—H = 0.95 Å and with *U*_iso_(H) = 1.5*U*_eq_(C) for methyl H or 1.2*U*_eq_(C) for other H. H atoms bonded to N atoms were refined isotropically.

### S2. Results and discussion

The anthranilic acid and 2-amino­benzamide are presented as key precursors for many families of organic compounds like the alkaloids which contain the quinazoline scaffolds. The latter continues to attract an expanded inter­est for therapeutic research due to their various pharmacological activities such as anti­microbial (Jantova *et al.*, 2004; Shi *et al.*, 2013), anti­tumorigenic (Kubo *et al.*, 2005), anti­fungal (Dandia *et al.*, 2005), anti­hyperglycemic (Ram *et al.*, 2003), anti­inflammatory (Gineinah *et al.*, 2002; Baba *et al.*, 1996), anti­tumor (Forsch *et al.*, 2002) and protein kinase inhibitor (Levitzky, 2003). Among the several synthetic ways to yield the quinazoline ring system the use of anthranilic acid derivatives as starting materials is attractive.

The title compound (Fig. 1) crystallizes with one molecule in the asymmetric unit. Both peptide bonds adopt a trans configuration. The dihedral angle between the two aromatic rings [C11–C16 and C21–C26] is 53.58 (4)°. The molecular conformation is stabilized by an intra­molecular N—H···O hydrogen bond (see Table 1). The crystal packing is characterized by zigzag chains of N—H···O hydrogen-bonded molecules running along the crystallographic b axis (Fig. 2).

### Crystal data

**Table:**

C_20_H_24_N_2_O_4_	*F*(000) = 760
*M**_r_* = 356.41	*D*_x_ = 1.288 Mg m^−^^3^
Monoclinic, *P*2_1_/*n*	Mo *K*α radiation, λ = 0.71073 Å
*a* = 8.4901 (4) Å	Cell parameters from 59538 reflections
*b* = 14.2281 (7) Å	θ = 3.6–29.9°
*c* = 15.3864 (7) Å	µ = 0.09 mm^−^^1^
β = 98.659 (4)°	*T* = 173 K
*V* = 1837.46 (15) Å^3^	Block, colourless
*Z* = 4	0.36 × 0.35 × 0.32 mm

### Data collection

**Table:**

Stoe IPDS II two-circle diffractometer	4676 reflections with *I* > 2σ(*I*)
Radiation source: Genix 3D IµS microfocus X-ray source	*R*_int_ = 0.065
ω scans	θ_max_ = 29.7°, θ_min_ = 3.6°
Absorption correction: multi-scan (*X-AREA*; Stoe & Cie, 2001)	*h* = −11→10
*T*_min_ = 0.874, *T*_max_ = 0.892	*k* = −19→19
56243 measured reflections	*l* = −21→21
5148 independent reflections	

### Refinement

**Table:**

Refinement on *F*^2^	Hydrogen site location: mixed
Least-squares matrix: full	H atoms treated by a mixture of independent and constrained refinement
*R*[*F*^2^ > 2σ(*F*^2^)] = 0.041	*w* = 1/[σ^2^(*F*_o_^2^) + (0.0525*P*)^2^ + 0.4445*P*] where *P* = (*F*_o_^2^ + 2*F*_c_^2^)/3
*wR*(*F*^2^) = 0.110	(Δ/σ)_max_ < 0.001
*S* = 1.08	Δρ_max_ = 0.35 e Å^−^^3^
5148 reflections	Δρ_min_ = −0.17 e Å^−^^3^
244 parameters	Extinction correction: *SHELXL97* (Sheldrick, 2008), Fc^*^=kFc[1+0.001xFc^2^λ^3^/sin(2θ)]^-1/4^
0 restraints	Extinction coefficient: 0.020 (2)

### Special details

**Table:**

Geometry. All e.s.d.'s (except the e.s.d. in the dihedral angle between two l.s. planes) are estimated using the full covariance matrix. The cell e.s.d.'s are taken into account individually in the estimation of e.s.d.'s in distances, angles and torsion angles; correlations between e.s.d.'s in cell parameters are only used when they are defined by crystal symmetry. An approximate (isotropic) treatment of cell e.s.d.'s is used for estimating e.s.d.'s involving l.s. planes.

### Fractional atomic coordinates and isotropic or equivalent isotropic displacement parameters (Å^2^)

**Table:**

	*x*	*y*	*z*	*U*_iso_*/*U*_eq_	
N1	0.62897 (11)	0.11518 (6)	0.37268 (6)	0.02663 (18)	
H1	0.6531 (19)	0.1522 (11)	0.4190 (10)	0.039 (4)*	
N2	0.73335 (10)	0.39881 (6)	0.34983 (5)	0.02395 (17)	
H2	0.7336 (17)	0.4150 (10)	0.2968 (10)	0.031 (3)*	
O1	0.67588 (12)	−0.02751 (6)	0.31368 (5)	0.0385 (2)	
O2	0.69514 (10)	0.28243 (5)	0.44371 (5)	0.02974 (17)	
O3	0.80069 (8)	0.55548 (5)	0.53889 (5)	0.02611 (16)	
O4	0.59596 (8)	0.56951 (5)	0.41902 (5)	0.02602 (16)	
C1	0.67865 (13)	0.02429 (7)	0.37787 (7)	0.0271 (2)	
C2	0.67557 (11)	0.31442 (7)	0.36759 (6)	0.02229 (18)	
C3	0.81688 (11)	0.45736 (7)	0.41942 (6)	0.02321 (18)	
H3A	0.8822	0.5043	0.3934	0.028*	
H3B	0.8898	0.4176	0.4602	0.028*	
C4	0.70242 (11)	0.50823 (7)	0.47073 (6)	0.02223 (18)	
H4	0.6387	0.4601	0.4976	0.027*	
C5	0.71605 (13)	0.59880 (9)	0.60186 (8)	0.0347 (2)	
H5A	0.6615	0.5504	0.6327	0.042*	
H5B	0.6347	0.6426	0.5721	0.042*	
C6	0.83490 (14)	0.65147 (8)	0.66656 (7)	0.0330 (2)	
H6A	0.7797	0.6819	0.7105	0.049*	
H6B	0.9146	0.6075	0.6958	0.049*	
H6C	0.8878	0.6993	0.6354	0.049*	
C7	0.66672 (14)	0.64600 (9)	0.37833 (9)	0.0375 (3)	
H7A	0.6954	0.6257	0.3212	0.045*	
H7B	0.7649	0.6671	0.4164	0.045*	
C8	0.54856 (18)	0.72486 (10)	0.36468 (11)	0.0473 (3)	
H8A	0.5950	0.7779	0.3368	0.071*	
H8B	0.4521	0.7035	0.3267	0.071*	
H8C	0.5213	0.7447	0.4216	0.071*	
C11	0.74040 (13)	−0.00903 (7)	0.46888 (7)	0.0277 (2)	
C12	0.84330 (16)	−0.08571 (8)	0.47741 (9)	0.0391 (3)	
H12	0.8685	−0.1164	0.4264	0.047*	
C13	0.90937 (19)	−0.11757 (10)	0.56026 (10)	0.0493 (3)	
H13	0.9823	−0.1686	0.5658	0.059*	
C14	0.86936 (19)	−0.07526 (11)	0.63444 (9)	0.0509 (4)	
H14	0.9139	−0.0975	0.6910	0.061*	
C15	0.76431 (18)	−0.00032 (10)	0.62657 (8)	0.0425 (3)	
H15	0.7361	0.0283	0.6779	0.051*	
C16	0.69998 (14)	0.03331 (8)	0.54417 (7)	0.0319 (2)	
H16	0.6285	0.0851	0.5391	0.038*	
C21	0.56160 (11)	0.16374 (7)	0.29641 (6)	0.02321 (18)	
C22	0.58342 (11)	0.26171 (7)	0.29212 (6)	0.02224 (18)	
C23	0.51100 (13)	0.30950 (7)	0.21728 (7)	0.0273 (2)	
H23	0.5245	0.3756	0.2136	0.033*	
C24	0.41991 (13)	0.26268 (8)	0.14820 (7)	0.0301 (2)	
H24	0.3724	0.2963	0.0976	0.036*	
C25	0.39886 (12)	0.16645 (8)	0.15357 (7)	0.0291 (2)	
H25	0.3359	0.1341	0.1067	0.035*	
C26	0.46886 (13)	0.11722 (7)	0.22676 (7)	0.0274 (2)	
H26	0.4538	0.0512	0.2297	0.033*	

### Atomic displacement parameters (Å^2^)

**Table:**

	*U*^11^	*U*^22^	*U*^33^	*U*^12^	*U*^13^	*U*^23^
N1	0.0370 (5)	0.0208 (4)	0.0209 (4)	−0.0003 (3)	0.0005 (3)	−0.0013 (3)
N2	0.0285 (4)	0.0242 (4)	0.0187 (4)	−0.0022 (3)	0.0021 (3)	−0.0009 (3)
O1	0.0569 (5)	0.0297 (4)	0.0287 (4)	0.0088 (4)	0.0053 (4)	−0.0057 (3)
O2	0.0428 (4)	0.0251 (3)	0.0196 (3)	−0.0033 (3)	−0.0007 (3)	0.0001 (3)
O3	0.0214 (3)	0.0309 (4)	0.0254 (3)	−0.0009 (3)	0.0015 (3)	−0.0082 (3)
O4	0.0204 (3)	0.0267 (3)	0.0302 (4)	0.0001 (3)	0.0013 (3)	0.0035 (3)
C1	0.0332 (5)	0.0230 (4)	0.0253 (4)	−0.0003 (4)	0.0053 (4)	−0.0003 (3)
C2	0.0241 (4)	0.0217 (4)	0.0204 (4)	0.0022 (3)	0.0013 (3)	−0.0020 (3)
C3	0.0213 (4)	0.0237 (4)	0.0241 (4)	−0.0017 (3)	0.0018 (3)	−0.0034 (3)
C4	0.0206 (4)	0.0228 (4)	0.0227 (4)	−0.0014 (3)	0.0015 (3)	−0.0015 (3)
C5	0.0279 (5)	0.0443 (6)	0.0321 (5)	−0.0004 (4)	0.0054 (4)	−0.0143 (5)
C6	0.0389 (6)	0.0293 (5)	0.0308 (5)	−0.0042 (4)	0.0051 (4)	−0.0078 (4)
C7	0.0303 (5)	0.0354 (6)	0.0472 (7)	0.0008 (4)	0.0070 (5)	0.0155 (5)
C8	0.0459 (7)	0.0348 (6)	0.0597 (8)	0.0072 (5)	0.0027 (6)	0.0140 (6)
C11	0.0333 (5)	0.0225 (4)	0.0268 (5)	−0.0040 (4)	0.0035 (4)	0.0026 (3)
C12	0.0471 (7)	0.0298 (5)	0.0412 (6)	0.0055 (5)	0.0093 (5)	0.0092 (5)
C13	0.0513 (8)	0.0415 (7)	0.0539 (8)	0.0059 (6)	0.0043 (6)	0.0229 (6)
C14	0.0593 (8)	0.0510 (8)	0.0384 (7)	−0.0101 (6)	−0.0055 (6)	0.0217 (6)
C15	0.0573 (8)	0.0435 (6)	0.0262 (5)	−0.0135 (6)	0.0042 (5)	0.0042 (5)
C16	0.0392 (6)	0.0288 (5)	0.0277 (5)	−0.0065 (4)	0.0048 (4)	−0.0001 (4)
C21	0.0257 (4)	0.0237 (4)	0.0204 (4)	0.0009 (3)	0.0039 (3)	−0.0019 (3)
C22	0.0238 (4)	0.0228 (4)	0.0197 (4)	0.0009 (3)	0.0021 (3)	−0.0021 (3)
C23	0.0311 (5)	0.0251 (4)	0.0240 (4)	0.0007 (4)	−0.0014 (4)	0.0003 (3)
C24	0.0307 (5)	0.0343 (5)	0.0231 (4)	0.0019 (4)	−0.0032 (4)	0.0002 (4)
C25	0.0271 (5)	0.0353 (5)	0.0238 (4)	−0.0023 (4)	0.0003 (4)	−0.0070 (4)
C26	0.0310 (5)	0.0254 (4)	0.0256 (4)	−0.0037 (4)	0.0034 (4)	−0.0051 (4)

### Geometric parameters (Å, º)

**Table:**

N1—C1	1.3589 (13)	C7—H7B	0.9900
N1—C21	1.4069 (12)	C8—H8A	0.9800
N1—H1	0.886 (16)	C8—H8B	0.9800
N2—C2	1.3406 (12)	C8—H8C	0.9800
N2—C3	1.4542 (12)	C11—C12	1.3915 (16)
N2—H2	0.848 (15)	C11—C16	1.3937 (15)
O1—C1	1.2296 (13)	C12—C13	1.3892 (18)
O2—C2	1.2441 (11)	C12—H12	0.9500
O3—C4	1.4088 (11)	C13—C14	1.377 (2)
O3—C5	1.4299 (13)	C13—H13	0.9500
O4—C4	1.4120 (11)	C14—C15	1.384 (2)
O4—C7	1.4319 (13)	C14—H14	0.9500
C1—C11	1.4965 (14)	C15—C16	1.3878 (16)
C2—C22	1.4998 (12)	C15—H15	0.9500
C3—C4	1.5240 (13)	C16—H16	0.9500
C3—H3A	0.9900	C21—C26	1.3973 (13)
C3—H3B	0.9900	C21—C22	1.4091 (13)
C4—H4	1.0000	C22—C23	1.3976 (13)
C5—C6	1.5061 (15)	C23—C24	1.3871 (14)
C5—H5A	0.9900	C23—H23	0.9500
C5—H5B	0.9900	C24—C25	1.3850 (16)
C6—H6A	0.9800	C24—H24	0.9500
C6—H6B	0.9800	C25—C26	1.3821 (15)
C6—H6C	0.9800	C25—H25	0.9500
C7—C8	1.4987 (17)	C26—H26	0.9500
C7—H7A	0.9900		
			
C1—N1—C21	126.87 (8)	H7A—C7—H7B	108.4
C1—N1—H1	119.1 (10)	C7—C8—H8A	109.5
C21—N1—H1	113.3 (10)	C7—C8—H8B	109.5
C2—N2—C3	121.06 (8)	H8A—C8—H8B	109.5
C2—N2—H2	119.5 (10)	C7—C8—H8C	109.5
C3—N2—H2	118.8 (10)	H8A—C8—H8C	109.5
C4—O3—C5	114.11 (7)	H8B—C8—H8C	109.5
C4—O4—C7	116.14 (8)	C12—C11—C16	119.35 (10)
O1—C1—N1	123.70 (10)	C12—C11—C1	117.59 (10)
O1—C1—C11	121.56 (9)	C16—C11—C1	123.06 (10)
N1—C1—C11	114.73 (9)	C13—C12—C11	120.23 (13)
O2—C2—N2	121.22 (9)	C13—C12—H12	119.9
O2—C2—C22	121.74 (9)	C11—C12—H12	119.9
N2—C2—C22	117.02 (8)	C14—C13—C12	120.12 (13)
N2—C3—C4	112.03 (8)	C14—C13—H13	119.9
N2—C3—H3A	109.2	C12—C13—H13	119.9
C4—C3—H3A	109.2	C13—C14—C15	120.03 (12)
N2—C3—H3B	109.2	C13—C14—H14	120.0
C4—C3—H3B	109.2	C15—C14—H14	120.0
H3A—C3—H3B	107.9	C14—C15—C16	120.36 (13)
O3—C4—O4	112.45 (8)	C14—C15—H15	119.8
O3—C4—C3	105.07 (7)	C16—C15—H15	119.8
O4—C4—C3	113.91 (8)	C15—C16—C11	119.87 (12)
O3—C4—H4	108.4	C15—C16—H16	120.1
O4—C4—H4	108.4	C11—C16—H16	120.1
C3—C4—H4	108.4	C26—C21—N1	121.23 (9)
O3—C5—C6	107.89 (9)	C26—C21—C22	119.70 (9)
O3—C5—H5A	110.1	N1—C21—C22	119.02 (8)
C6—C5—H5A	110.1	C23—C22—C21	118.38 (9)
O3—C5—H5B	110.1	C23—C22—C2	120.54 (8)
C6—C5—H5B	110.1	C21—C22—C2	121.02 (8)
H5A—C5—H5B	108.4	C24—C23—C22	121.54 (9)
C5—C6—H6A	109.5	C24—C23—H23	119.2
C5—C6—H6B	109.5	C22—C23—H23	119.2
H6A—C6—H6B	109.5	C25—C24—C23	119.41 (9)
C5—C6—H6C	109.5	C25—C24—H24	120.3
H6A—C6—H6C	109.5	C23—C24—H24	120.3
H6B—C6—H6C	109.5	C26—C25—C24	120.41 (9)
O4—C7—C8	108.32 (10)	C26—C25—H25	119.8
O4—C7—H7A	110.0	C24—C25—H25	119.8
C8—C7—H7A	110.0	C25—C26—C21	120.56 (9)
O4—C7—H7B	110.0	C25—C26—H26	119.7
C8—C7—H7B	110.0	C21—C26—H26	119.7
			
C21—N1—C1—O1	2.80 (18)	C13—C14—C15—C16	−0.7 (2)
C21—N1—C1—C11	−177.98 (9)	C14—C15—C16—C11	0.47 (19)
C3—N2—C2—O2	−1.91 (14)	C12—C11—C16—C15	0.99 (17)
C3—N2—C2—C22	176.69 (8)	C1—C11—C16—C15	−178.76 (11)
C2—N2—C3—C4	−78.73 (11)	C1—N1—C21—C26	31.59 (16)
C5—O3—C4—O4	62.87 (11)	C1—N1—C21—C22	−150.91 (10)
C5—O3—C4—C3	−172.70 (9)	C26—C21—C22—C23	−0.19 (14)
C7—O4—C4—O3	57.49 (11)	N1—C21—C22—C23	−177.73 (9)
C7—O4—C4—C3	−61.91 (11)	C26—C21—C22—C2	176.98 (9)
N2—C3—C4—O3	175.66 (8)	N1—C21—C22—C2	−0.56 (14)
N2—C3—C4—O4	−60.83 (10)	O2—C2—C22—C23	155.43 (10)
C4—O3—C5—C6	−176.28 (9)	N2—C2—C22—C23	−23.16 (13)
C4—O4—C7—C8	−153.25 (10)	O2—C2—C22—C21	−21.68 (14)
O1—C1—C11—C12	21.90 (16)	N2—C2—C22—C21	159.73 (9)
N1—C1—C11—C12	−157.34 (10)	C21—C22—C23—C24	−0.21 (15)
O1—C1—C11—C16	−158.35 (11)	C2—C22—C23—C24	−177.39 (10)
N1—C1—C11—C16	22.41 (15)	C22—C23—C24—C25	0.54 (16)
C16—C11—C12—C13	−2.25 (18)	C23—C24—C25—C26	−0.49 (17)
C1—C11—C12—C13	177.51 (12)	C24—C25—C26—C21	0.10 (16)
C11—C12—C13—C14	2.1 (2)	N1—C21—C26—C25	177.72 (10)
C12—C13—C14—C15	−0.6 (2)	C22—C21—C26—C25	0.24 (15)

### Hydrogen-bond geometry (Å, º)

**Table:**

*D*—H···*A*	*D*—H	H···*A*	*D*···*A*	*D*—H···*A*
N1—H1···O2	0.886 (16)	1.914 (15)	2.6434 (11)	138.6 (14)
N2—H2···O1^i^	0.848 (15)	2.132 (15)	2.9338 (12)	157.6 (13)

Symmetry code: (i) −*x*+3/2, *y*+1/2, −*z*+1/2.
