# Supplementary figures and images for: Crystal structure of 2-benzamido-N-(2,2-di­eth­oxy­eth­yl)benzamide
Source: Acta Crystallogr E Crystallogr Commun. 2015 Feb 28;71(Pt 3):o214–5. doi: 10.1107/S2056989015003370 (PMC4350703; doi:10.1107/S2056989015003370)

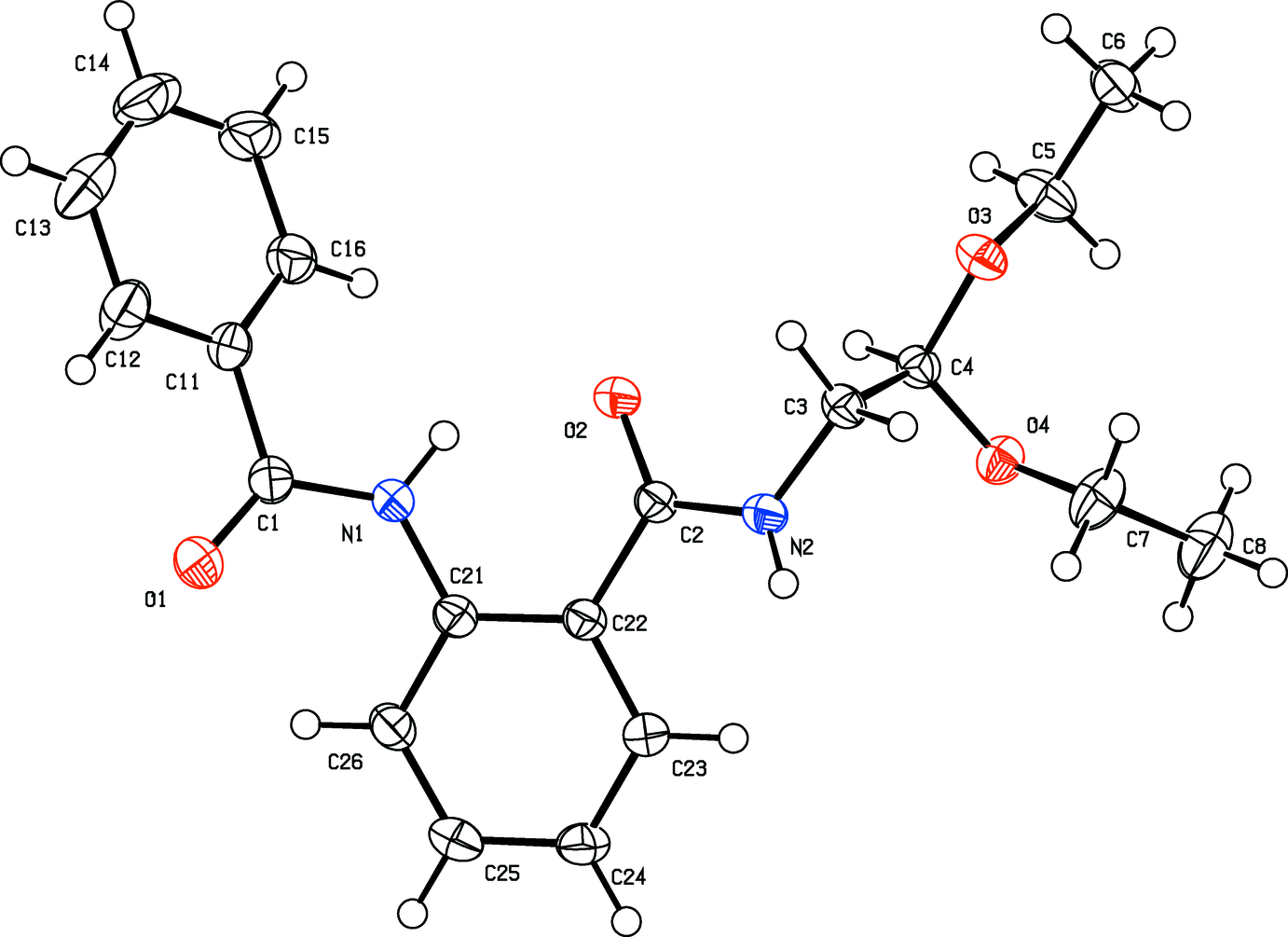

Supplement: Supplementary file 4 [file e-71-0o214-fig1.tif]

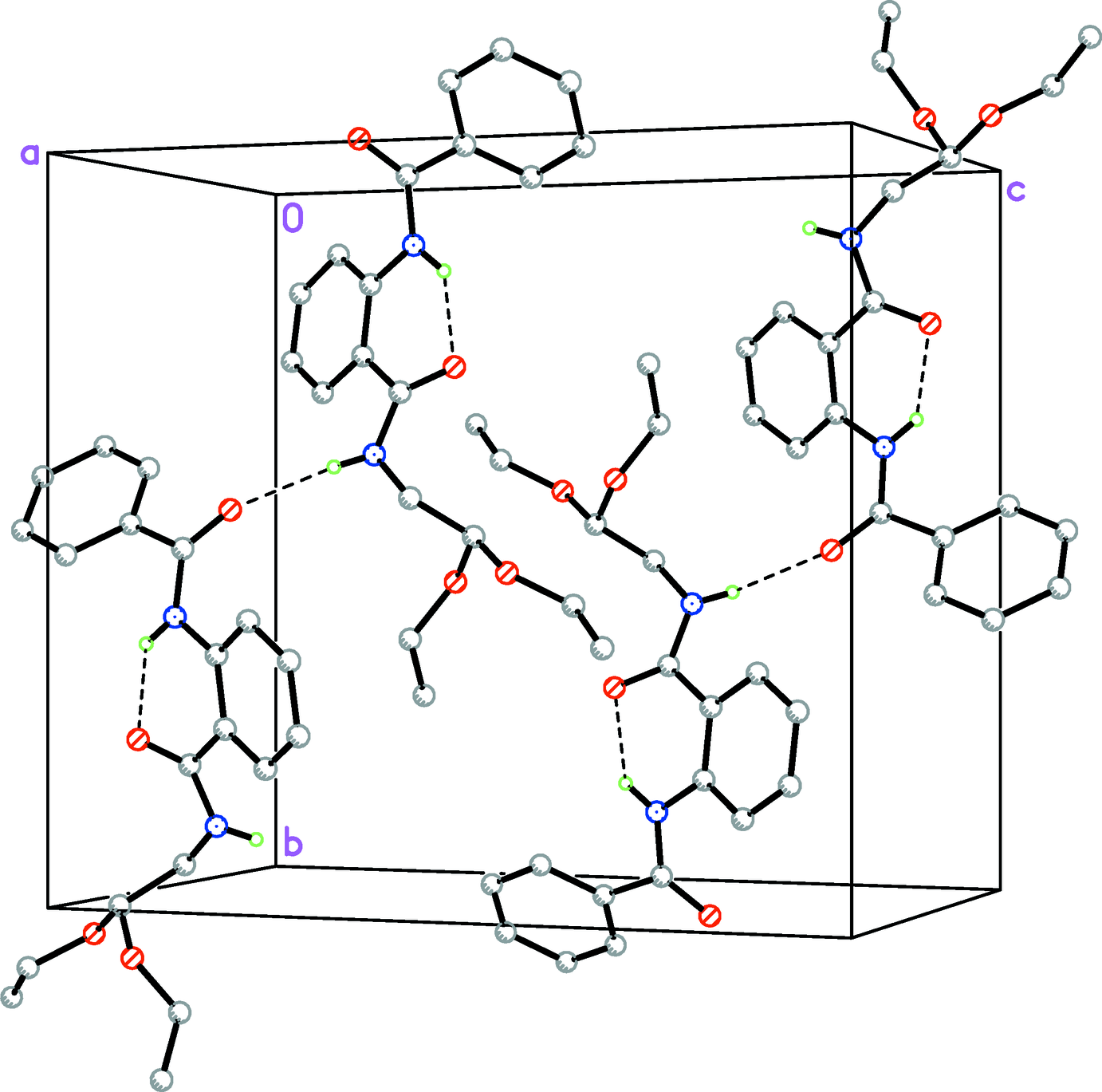

Supplement: Supplementary file 5 [file e-71-0o214-fig2.tif]
